# Comparison of three regimens with inhalational methoxyflurane versus intranasal fentanyl versus intravenous morphine in pre-hospital acute pain management: study protocol for a randomized controlled trial (PreMeFen)

**DOI:** 10.1186/s13063-023-07590-9

**Published:** 2023-09-05

**Authors:** Randi Simensen, Lars Olav Fjose, Marius Rehn, Jostein Hagemo, Kjetil Thorsen, Fridtjof Heyerdahl

**Affiliations:** 1 Division of Pre-Hospital Services, Innlandet Hospital Trust, Kastbakkvegen 9, 2390 Moelv, Norway; 2 Department of Research, Norwegian Air Ambulance Foundation, Oslo, Norway; 3 Division of Pre-Hospital Services, Oslo University Hospital, Oslo, Norway; 4 Faculty of Medicine, Institute of Clinical Medicine, University of Oslo, Oslo, Norway

**Keywords:** Analgesia, Pre-hospital, Emergency medicine, Acute pain, Opioids, Methoxyflurane

## Abstract

**Background:**

Pre-hospital pain management has traditionally been performed with intravenous (IV) morphine, but oligoanalgesia remain a recognized problem. Pain reduction is essential for patient satisfaction and is regarded as a measure of successful treatment. We aim to establish whether non-invasive methods such as inhalation of methoxyflurane is non-inferior to intranasal fentanyl or non-inferior to the well-known IV morphine in the pre-hospital treatment of acute pain.

**Method/design:**

The PreMeFen study is a phase three, three-armed, randomized, controlled, non-inferiority trial to compare three regimens of analgesics: inhalation of methoxyflurane and intranasal (IN) fentanyl versus IV morphine. It is an open-label trial with a 1:1:1 randomization to the three treatment groups. The primary endpoint is the change in pain numeric rating scale (NRS) (0–10) from baseline to 10 min after start of investigational medicinal product administration (IMP). The non-inferiority margin was set to 1.3, and a sample size of 270 patients per protocol (90 in each treatment arm) will detect this difference with 90% power.

**Discussion:**

We chose a study design with comparison of analgesic regimens rather than fixed doses because of the substantial differences in drug characteristics and for the results to be relevant to inform policymakers in the pre-hospital setting. We recognize that easier administration of analgesics will lead to better pain management for many patients if the regimens are as good as the existing, and hence, we chose a non-inferiority design. The primary endpoint, the change in pain (NRS) after 10 min, is set to address the immediate need of pain reduction for patients with acute prehospital pain. On a later stage, more analgesic methods are often available.

**Summary:**

PreMeFen is a non-inferiority randomized controlled trial comparing three analgesic regimens aiming to establish whether inhalation of methoxyflurane or intranasal fentanyl is as good as IV morphine for fast reduction of acute pain in the prehospital setting.

**Supplementary Information:**

The online version contains supplementary material available at 10.1186/s13063-023-07590-9.

## Background

Pre-hospital underuse of analgesics (oligoanalgesia) remains a global health problem [1–4]. Between 35 and 53% of patients describe moderate to severe pain during pre-hospital management [5], and pain management is considered a primary task for emergency medical service (EMS) providers [6, 7]. Still, barriers to adequate analgesia include challenging environment, lack of competence, and experience [8]. Although pre-hospital pain management often involves IV opioid administration [9], cannulation has been described to fail in 12–26% of adults [10], leading to a potential delay in administration of analgesia. Patients may benefit from a more easy-to-administer analgetic, which should be delivered safely, effectively, non-invasively and should be fast-acting. Reviews suggest two such alternatives: IN fentanyl, a synthetic opioid [8], and inhaled low-dose methoxyflurane, a volatile anesthetic from a group of fluorinated hydrocarbons [11]. Methoxyflurane in low dose is a non-narcotic analgesic widely used in Australia and New Zealand [11]. The slightly volatile gas is administered as a liquid via an inhalation chamber in a whistle-like pipe, while the exhaled air is filtered in a coal chamber on the same whistle [12].

## Study rationale

The rationale of the PreMeFen-study is to provide evidence for earlier, safe, non-invasive pain management in the pre-hospital setting.

We aim to investigate whether regimens of:

- Inhaled low-dose methoxyflurane is non-inferior to IN fentanyl or
- Inhaled low-dose methoxyflurane is non-inferior to IV morphine or
- IN fentanyl is non-inferior to IV morphine

in managing a variety of moderate to severe acute pain conditions in adult patients from 18 years of age administered by the EMS.

## Study setting

Norway has a governmentally funded healthcare system aiming to provide equal access to healthcare regardless of geographical location and resource availability. The Ministry of Health and Care Services carries supervisory responsibility for all the public hospitals and is the owner of four regional health trusts [13]. The ambulance services are integrated in Norwegian specialist health service system [14–16]. This study will be conducted at the Innlandet Hospital Trust, Pre-Hospital Division (Norway), ground ambulances service. The service runs 44 ambulances with a catchment area of approximately 52,000 square kilometers and 360,000 inhabitants in rural and urban areas. Three ambulance stations were selected to include patients, of which two are based in cities (Gjøvik and Lillehammer), whereas one is rural (Gran). Selection was based on activity data to ensure adequate subject recruitment. The emergency medical communication center (EMCC) manages emergency medical calls from the public. EMCC is staffed with trained health care personnel (paramedics and nurses) using Norwegian Index for Emergency Medical Assistance (INDEX), a criteria-based medical dispatch guideline [17].

## Methods

### Study design

We applied the Norwegian Clinical Research Infrastructure Network (NorCRIN) templates [18] to design a randomized, controlled, open-label, three-arm, non-inferiority, phase three drug trial. The trial will be performed according to the principles of the Helsinki Declaration. The randomization is 1:1:1 to the three treatment groups, with 90 patients in each group, totaling 270 patients. Patients in all treatment groups follow a treatment regime rather than receiving a single drug dose. For ethical reasons, a placebo arm was excluded. The study protocol was drafted in accordance with the Standard Protocol Items: Recommendations for Interventional Trial guidelines (SPIRIT) [19].

### Allocation and randomization

**Fig. 1:**
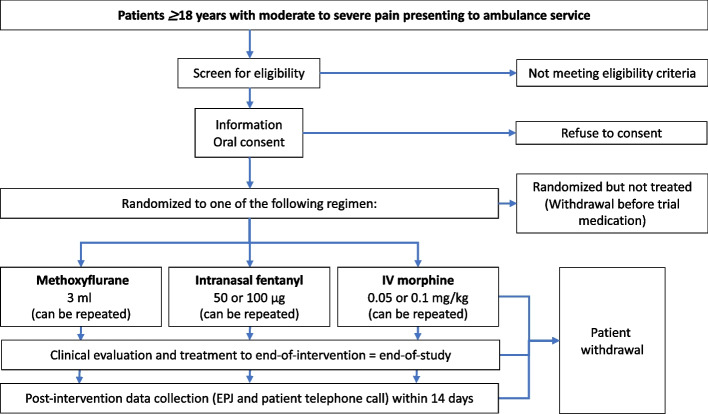
Flow chart study intervention

The study kit contains all three IMP, patient information letter, information letter to receiving hospital, and a sealed randomization envelope. Computer-generated block randomization with variable block sizes is provided by the department of clinical trial unit (CTU), Oslo University Hospital. All the sealed randomization envelopes were provided in one batch from the CTU, which also kept the code file secret and hidden from the study team. The sealed envelopes were further distributed into the study kits by the study team. Following routine clinical primary survey, the EMS providers screen for eligibility and ask for consent. The study kit remains sealed until the patient is included, and the consent form is signed by the EMS provider. Patients are then randomized 1:1:1 between the three treatment regimes, as shown in Fig. 1.

### Blinding

General blinding was considered unfeasible due to the complexity of study context, but the datasets is blinded for the statistician.

### Eligibility criteria

EMCC does not pre-screen patients, making eligibility evaluation solely to be performed on-scene. Patients considered not eligible are treated according to local analgesic protocols. See Table 1 for inclusion and exclusion criteria.

**Table 1:** Inclusion and exclusion criteria

Inclusion criteria	Exclusion criteria
> 18 years of age	Life-threatening or limb-threatening condition requiring immediate management
Acute moderate to severe pain (both medical and traumatic etiology) defined by self-reporting pain ≥ 4 on numeric rating scale	Pregnancy or breastfeeding
Capable of giving informed consent	Know allergies, hypersensitivity, or serious side effects to opioids or methoxyflurane or other excipients
Normal physiology	Head injury or medical conditions with neurological impairment (Glasgow coma scale < 14)
	Previous malignant hyperthermia or persons with suspect genetic predisposition for malignant hyperthermia
	Massive facial trauma, visible nasal blockage, or on-going nose bleeding
	History of severe liver disease with jaundice and scleral icterus
	Dialysis or history of severe renal disease (known chronic kidney failure stage 4 or 5)
	Monoaminoxidase inhibitors last 14 days (pharmacological treatment of depression, Morbus Parkinson or narcolepsy)
	Myasthenia gravis
	Use of investigational medicinal product analgesics 12 h prior to inclusion
	Any condition that in the view of the study worker would suggest that the patient is unable to comply with study protocol and procedures

### Interventions

The intervention is the administration of IMP with three different medication regimens of inhalational methoxyflurane, IN fentanyl, and IV morphine; see Table 2. The schedule of activities is outlined in Table 3. After inclusion, baseline clinical data is obtained. Clinical data consists of NRS, Glasgow coma scale (GCS) [20], heart rate, blood pressure, oxygen saturation, and respiratory rate. IMP is given at T0. NRS is repeated after 5 min. All clinical data is repeated after 10, 20, and 30 min and upon arrival in emergency department, whatever comes first. The patient may receive additional doses of IMP from 5 min after first dose. The study duration is from ambulance scene arrival to patient handover in the emergency department (ED).

**Table 2:** Medication regimens

IMP	Methoxyflurane	IN fentanyl	IV morphine
Dose	3 ml inhalation	18–70 years: 100 μg > 70 years: 50 μg	18–70 years: 0.1 mg/kg > 70 years or fragile: 0.05 mg/kg
Dose repetition	Yes, if needed	Yes, if needed interval 5 min	Yes, if needed interval 5 min
Maximum total dose	6 ml	500 μg	0.5 mg/kg

*IN* Intranasal, *IV* Intravenous, *IMP* Investigational medicinal product

**Table 3:** Schedule of activities

Timepoint	T(x)	T_0_	T_5_	T_10_	T_20_	T_30_	T_ED_	Within 14 days
Description	Scene arrival	IMP adm	5 min	10 min	20 min	30 min	Arrival ED	
Inclusion and exclusion criteria	X							
Informed oral consent	X							
Physical examination including estimation of weight	X							
Medical history	X							
Allocation	X							
ECG (If chest pain)	(X)						(X)	
Intravenous access attempts	X							
Start administration of IMP		X						
SpO_2_	X			X	X	X	X	
Blood pressure	X			X		X	X	
Heart rate	X			X	X	X	X	
Respiration rate	X			X		X	X	
NRS	X		X	Primary endpoint	X	X	X	
GCS	X			X		X	X	
Patient satisfaction Likert scale							X	
HCP satisfaction Likert scale							X	
Troponin I/T							X	
Recording final diagnosis								X

*IMP* Investigational medicinal product, *ECG* Electrocardiogram, *SpO*_*2*_ Oxygen saturation, *NRS* Numeric rating scale, *GCS* Glasgow come scale, *HCP* Health care personnel

### Medication regimens

Administration of each of the 3 IMPs is based on pre-specified treatment regimens, including a flexible dosing of the analgesic and titration to effect, but only using the specific drug in the allocated treatment arm. Each regimen is defined with doses, dose intervals, and maximum doses (see Table 2 for medication regimes).

### Rescue medication

In cases where IMP fail to provide sufficient analgesic effect (NRS < 4) despite repeated doses, patients are treated with standard pain relief protocol. All analgesics other than the allocated IMP is referred to as rescue medication. Any need for rescue medication is noted with time and dose.

In cases where rescue medication is administered before the primary endpoint assessment at 10 min, the patient will be excluded from the per-protocol analysis.

### NRS pain score scale

NRS is a scale with integer values from 0 to 10 where 0 indicates no pain and 10 indicates unbearable pain. Patients indicate on a line with numbers ranging from 0 to 10 or verbally, what numbered value the pain correlates to, and the score is documented in the case report form (CRF).

### Endpoints

Primary endpoint is the change in NRS pain score measured prior to administration of IMP to 10 min after IMP administration; see Table 4 for secondary and exploratory endpoints.

**Table 4:** Study endpoints

Primary endpoint	• Changes in pain score from T_0_ to T_10_
Secondary endpoints	• Changes in pain score from T_0_ to T_5_, T_20_, T_30_ and end of mission T_ED_ • Need for additional analgesia not in the regimen of the allocated treatment group • Differences in time arrival to administration of IMP registration of AE and SAE during study period until end of intervention • Time from ambulance personnel arrival to first measure > 2 points reduction in NRS from baseline • Change in level of sedation from T_0_ to T_10_ and T_30_ • Change in RR T_0_ to T_10_ and T_30_ • Change in SBP T_0_ to T_10_ and T_30_ • Likert scale of HCP satisfaction at end of mission • Likert scale of patient satisfaction at end of mission • Registration of AE and SAE during study period until end of intervention
Exploratory endpoints	• Analyze primary and secondary efficacy endpoints stratified by diagnosis or diagnosis groups • Proportion of patient receiving rescue treatment related to procedures (reposition of fractures, relocation, etc.) • Attempts of vascular cannulation access • Change in NRS and time to a significant NRS reduction compared to level of competence • Ambulance personnel competence and patient satisfaction • Analyze primary and secondary efficacy endpoints stratified by level of troponin after ED admission and sign of ACS on ECG at scene • Analyze AE and SAE in relation to concomitant therapy and other non-IMP determinants

*IMP* Investigational medicinal product, *AE* Adverse event, *SAE* Severe adverse event, *NRS* Numeric rating scale, *RR* Respiratory rate, *SBP* Systolic blood pressure, *HCP* Health care personnel, *ED* emergency department, *ACS* Acute coronary syndrome, *ECG* Electrocardiogram, *T*_*-x*_ Scene arrival, *T*_*0*_ Time of IMP administration, *T*_*5*_ 5 min after IMP administration, *T*_*10*_ 10 min after IMP administration, *T*_*20*_ 20 min after IMP administration, *T*_*30*_ 30 min after IMP administration, *T*_*ED*_ Time arrival emergency department or end of service

### Hypotheses

The primary hypotheses describe the comparisons of the primary endpoints for the three different analgesic regimens:

1 $$\begin{array}{ccc}{\mathrm{H}}_{1}:& {h}_{01}: {\mathrm{m}}_{\mathrm{met}}-{\mathrm{m}}_{\mathrm{fen }}\le {\updelta }_{\mathrm{I}}, & {h}_{\mathrm{a}1}: {\mathrm{m}}_{\mathrm{met}}-{\mathrm{m}}_{\mathrm{fen}}> {\updelta }_{\mathrm{I}}\\ {\mathrm{H}}_{2}:& {h}_{02}:{\mathrm{m}}_{\mathrm{met}}-{\mathrm{m}}_{\mathrm{mor}} \le {\updelta }_{\mathrm{I}},& {h}_{\mathrm{a}2}: {\mathrm{m}}_{\mathrm{met}}-{\mathrm{m}}_{\mathrm{mor}}> {\updelta }_{\mathrm{I}}\\ {\mathrm{H}}_{3}:& {h}_{03}:{\mathrm{m}}_{\mathrm{fen}}-{\mathrm{m}}_{\mathrm{mo}}\mathrm{r }\le {\updelta }_{\mathrm{I}},& {h}_{\mathrm{a}3}: {\mathrm{m}}_{\mathrm{fen}}-{\mathrm{m}}_{\mathrm{mor}}> {\updelta }_{\mathrm{I}}\end{array}$$

where *m*_x_ is the mean reduction in NRS for treatment *x* and ${\delta }_{I}$ is the non-inferiority margin.

*Null hypothesis h*_*01*_*:* methoxyflurane regimen is inferior to IN fentanyl regimen.

*Null hypothesis *${h}_{02}$*:* methoxyflurane regimen is inferior to IV morphine regimen.

*Null hypothesis *${h}_{03}$*:* IN fentanyl regimen is inferior to the IV morphine regimen.

*Alternative hypothesis *${h}_{a1}$*:* methoxyflurane regimen is non-inferior to IN fentanyl regimen.

*Alternative hypothesis *${h}_{a2}$*:* methoxyflurane regimen is non-inferior to IV morphine regimen.

Alternative hypothesis ${h}_{a3}$: IN fentanyl regimen is non-inferior to IV morphine regimen.

### Sample size

The sample size with $\alpha$ = 0.05 and $\beta$ = 0.10 (90% power) was estimated using a two-sided *t*-test [11, 21, 22]. Expected pain reduction after 10 min was set to 3.77 for methoxyflurane, 2.54 for fentanyl, and 2.70 for morphine treatment regimen based on similar studies [11, 19, 20]. A common conservative standard variation of 2.20 was used. Non-inferiority margin is set to ${\delta }_{I}$ = 1.3 based on [22, 23]. Sample size required to detect this difference 10 min after administration of IMP was estimated to be *n* = 88 in each arm. That gives a total number of participants of 264, and the plan is to include 3 × 90 = 270 patients per protocol allowing for two dropouts in each group, in total six dropouts, of the per-protocol population without losing power for the calculations of the primary endpoint.

### Statistics

**Fig. 2:**
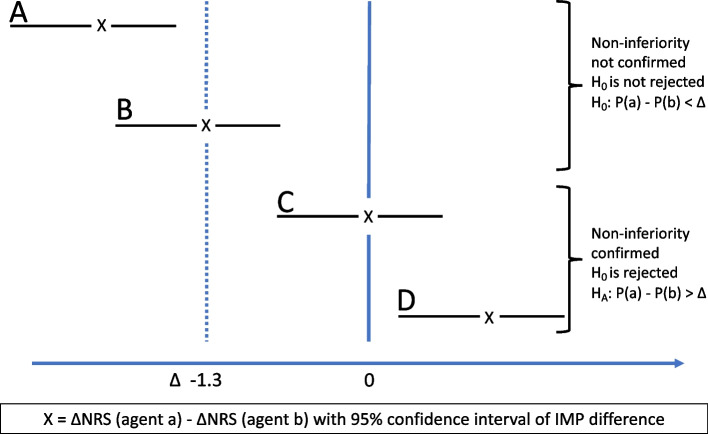
Non-inferiority chart. The lines A–D represent mean and confidence interval of the differences in NRS-changes in the two treatment groups a and b. Examples A and B show that the agent a is inferior to agent b, while examples C and D confirm that agent a is non-inferior to agent b. IMP, investigational medicinal product; NRS, numeric rating scale

The hypotheses in Eq. (1) will be tested and a conclusion of non-inferiority will be made if the 95% CI of estimated treatment difference fully lie above the inferiority margin; see Fig. 2. Hypotheses ${H}_{1}$, ${H}_{2}$, and ${H}_{3}$ will be tested using the fixed-sequence procedure, to avoid inflating the significant level for the overall test. Accordingly, family-wise error rate will remain the same as the local nominal significance level *a*. The test will be performed in the following order:

$${H}_{1}\to {H}_{2}\to {H}_{3}$$

The statistical chain of testing and decisions will be:

- *Test *${H}_{1}$*:* If part of the 95% confidence interval is below non-inferiority margin, we stop testing without any conclusion on efficacy. If the 95% confidence interval is above the non-inferiority margin, then we claim non-inferiority of methoxyflurane vs fentanyl and proceed with testing ${H}_{2}$.
- *Test *${H}_{2}$*:* If part of the 95% confidence interval is below the non-inferiority margin, we stop testing without any further conclusion on efficacy. If the 95% confidence interval is above the non-inferiority margin, then we claim non-inferiority of methoxyflurane vs morphine and proceed with testing ${H}_{3}$.
- *Test *${H}_{3}$*:* If part of the 95% confidence interval is below the non-inferiority margin, we stop testing without any further conclusion on efficacy. If the 95% confidence interval is above the non-inferiority margin, then we claim non-inferiority of fentanyl vs morphine.

The nominal significance level is set to 5%, and the non-inferiority margin is set to 1.3. Non-inferiority is determined based on a 1-sided equivalence *t*-test on the per protocol population and confirmed, for sensitivity reasons, on the modified intention to treat population.

We plan to compare the incidence of serious and non-serious adverse events between the groups using a chi-squared test or a Fisher exact test if necessary (expected frequency less than 5). We will summarize patient satisfaction and medical personnel view on practicality.

Demographic and baseline information of the three study groups will be compared using *t*-tests (means), Mann–Whitney *U* (medians), and chi-square (proportions) tests. If there are any significant differences, linear regression will be performed to adjust for significantly different covariates. No interim analysis will be performed.

### Data collection

Data are collected from ambulance records with study specific CRF, including hospital records and follow-up call to the patients 14 days after inclusion. In addition to IMP, the study kit contains a stopwatch and an EtCO2 nasal cannula. The investigators manually enter data into the electronic data capture software Viedoc (Viedoc Technologies AB, Uppsala, Sweden). All study-related information will be securely stored at the study site.

### Missing data

For non-inferiority calculation of the primary endpoint, missing data will be replaced using imputation. We will perform a linear regression adjustment for baseline pain.

Due to the pre-hospital circumstances with patients in pain and a situation of urgency, some missing data are to be accepted. The principal investigator is responsible for deciding whether participants with missing data are evaluable. Missing data are to be recorded and reported to maintain transparency.

### Data monitoring

A data monitoring plan is established and describes regular reviews of the CRFs for accuracy and completeness. According to the data management process, a specific data handling report will be made after the closing of the database.

### Data monitoring committee

An independent data monitoring committee (DMC) and safety board is established and governed by its charter. The DMC reviews recruitment, safety, protocol deviation, and adverse events with a session when half of the patients are included.

### Safety procedures

In case of overdosage of IN fentanyl or IV morphine, standard treatment procedure will be initiated, including IN or intramuscular (IM) naloxone, physical stimulation to keep patient awake, and respiratory support as indicated. Because the participant administers the methoxyflurane themselves, overdose symptoms such as drowsiness will self-limit when the administration whistle is not actively held in the mouth and sealed with the lips. Nevertheless, an overdose of methoxyflurane will be treated according to protocols for patients with drug overdose.

We established a study telephone number to use for patients who want to withdraw from study and for EMS providers to report serious adverse events (SAE). Study kit management with control and return of IMP is administrated by the study group.

### Training of study personnel

A mandatory 8-h training program is required to qualify as certified study personnel. The training consists of three parts: a practical simulation, a theory part, and a workshop with the study group.

### Adverse event

EMS provider will continuously describe any adverse events (AE) in the CRF. The monitoring of vital signs will detect clinical adverse events in addition to general questions and physical examination usual to the pre-hospital treatment situation. AEs will be coded according to the Medical Dictionary for Regulatory Activities (MedDRA) system by investigator. For each participant, the standard time for collecting and recording AE and serious adverse event (SAE) will be from first IMP dose until end of intervention. EMS providers must report SAE to the principal investigator (PI) within 24 h. If the SAE is considered to be a suspected unexpected serious adverse reaction (SUSAR), a report will be sent to the Norwegian Medicines Agency within a maximum of 7 days for fatal or life-threatening SUSARS and within maximum 15 days for other SUSARS from first knowledge of sponsor. The EMS provider can use a dedicated telephone number to contact a member of the study working group if they have any questions or concern about an AE or possible SAE.

## Discussion

The study rationale is to provide evidence for early, safe, and effective pain management in EMS with non-invasive and fast-acting analgesics.

We aim to compare regimens rather than a fixed dose of the medicaments. With regimen we mean a flexible dosing of the allocated analgesic by titrating to effect, but only administer the allocated IMP. There are several reasons for comparing regimens rather than a fixed dose. Firstly, the route of administration differs to a large extent, with the inhalation of methoxyflurane being continuous depending on the patient needs, versus bolus dosing of the others. In addition, pharmacokinetics with bioavailability, *C*_max_, and *T*_max_ are different for all three IMPs. Furthermore, clinical needs and pain characteristics are heterogenous with individual needs for titration and redosing that cannot be foreseen and hence should be tailored with redosing within the allocated regimen. Finally, comparison of regimens will address the clinical setting where the interesting objective is to find whether the regimens of non-invasive methods are non-inferior to the existing IV morphine procedure. This will make the results relevant for decision-makers when implementing new procedures in pre-hospital pain management.

Our choice of a non-inferiority trial design is based on the expectation that IN fentanyl and inhalation of low-dose methoxyflurane are as good as (non-inferior to) IV morphine and that the first two will contribute to an earlier and more practical administration of the analgesics in the pre-hospital setting. Furthermore, it is of great interest to establish whether the self-administrated inhalation of methoxyflurane is as good as IN fentanyl, because it would ease the pain management in many acute pre-hospital settings.

The rationale for selecting the primary endpoint is based on valid outcome measures gained from previous research [22, 24–26]. Mean difference in NRS at 15 and 30 min from the first administration of pain treatment is the primary outcome in these studies. We consider the greatest potential advantages for non-invasive analgesics to be at the beginning of the treatment, before IV access is established, and to enable evacuation and transport. Therefore, the most interesting time point for comparison should be early, and thus 10 min after initiation of treatment was chosen. NRS is a validated research tool for pain assessment and considered the optimal scale for evaluating pain among adult patients without cognitive impairment [27]. The European Medicines Agency *Guideline on the clinical development of medical products intended for the treatment of pain* state that pain self-assessment is the most valid measure of pain assessment [28]. Studies suggest that an NRS difference of 1.3 is a clinically significant difference [22, 26, 29, 30]. Therefore, the primary endpoint of change in NRS is set with a noninferiority margin of 1.3.

## Limitations

The non-blinding aspect is a limitation of the study and carries a risk that administration method can influence the patients or EMS providers preferences. Methoxyflurane has a distinct odor, and the routes of administration are entirely different in the three arms and impossible to blind without dummies. The only way to double-blind the study would be to provide a triple-dummy procedure, which is considered too complex and not feasible in this study context. On the contrary, the administration method can itself be considered part of the treatment; for example, the self-administering of methoxyflurane can give the patient a feeling of control with a positive impact on the pain treatment. Our open-label design without dummies will include these aspects in the results and can represent real life to a more significant extent.

In spite that self-assessment of pain is considered as the most valid pain-assessment for this kind of studies, NRS is a one-dimensional scale and does not describe the whole perspective of pain [27, 31].

To avoid inflating the significant level for the overall test, we chose a statistical plan to test the hypotheses by using fixed-sequence procedures. If the study fails to prove a non-inferiority in the first test (methoxyflurane to fentanyl), this will terminate the subsequent tests comparing methoxyflurane to morphine and fentanyl to morphine. The subsequent tests could in that case only be performed as exploratory tests with less impact.

## Conclusion

PreMeFen is a non-inferiority randomized controlled study comparing three analgesic regimens aiming to establish whether inhalation of methoxyflurane or IN fentanyl is as good as IV morphine for fast reduction of pain in the prehospital setting.

## Trial status

This article is based on protocol 3.0, dated 2 September 2021. The Study is ongoing, and patient recruitment started on 12 November 2021. We estimate an 18-month recruitment period; the estimated date for completed recruitment is April 2023.

## Dissemination

The results of the study will be published in per-reviewed medical journals.

### Supplementary Information

**Additional file 1.** SPIRIT Checklist.

## Data Availability

The full protocol, dataset, details of the statistical analysis plan, and statistical codes can be made accessible for researchers with non-commercial projects by contacting the corresponding author or project leader.

## Abbreviations

AE: Adverse event
DMC: Data monitoring committee
GCS: Glasgow Coma Scale
CRF: Case report form
ED: Emergency department
EDC: Electronic data capture
EMCC: Emergency medical communication center
EMS: Emergency Medical Services
IM: Intramuscular
IMP: Investigational medicinal product
IN: Intranasal
INDEX: Norwegian Index for Emergency Medical Assistance
IV: Intravenous
LEC: Local emergency center
IM: Intramuscular
NRS: Numeric rating scale
PI: Principal investigator
SAE: Serious adverse event
SOP: Standard operating procedure
SUSAR: Suspected unexpected serious adverse reaction
